# Supplementary material for: Contrasting patterns of divergence at the regulatory and sequence level in European Daphnia galeata natural populations
Source: Ecol Evol. 2019 Feb 12;9(5):2487–504. doi: 10.1002/ece3.4894 (PMC6405927; doi:10.1002/ece3.4894)
Supplement: Supplementary file 1 [file ECE3-9-2487-s001.docx]

Supplementary Figure 1: Gene Expression PCA for the first three principal components. Gene expression PCA of the four sampled populations for the first three prinicpal components pairwise as a matrix; Pop.G (Lake Greifensee), Pop.J (Jordan Reservoir), Pop.LC (Lake Constance) and Pop.M (Lake Müggelsee). Percentages on the X- and Y-axis indicate the percentage of variance explained by each principal component.

Figure S1

Supplementary Figure 2: SNP PCA for the first three principal components. SNP PCA of the four sampled populations for the first three prinicpal components pairwise as a matrix; Pop.G (Lake Greifensee), Pop.J (Jordan Reservoir), Pop.LC (Lake Constance) and Pop.M (Lake Müggelsee). Percentages on the X- and Y-axis indicate the percentage of variance explained by each principal component.

Figure S2

Supplementary Figure 3: Functional annotation. Pie charts showing the number of transcripts annotated. The outer circle represents the data for all 32903 transcripts. The inner circle represents the data for the differentially expressed transcripts. (a) Pie chart for (a) BLAST; (b) Pfam (c) orthoMCL

Figure S3
