# Supplementary figures and images for: Contrasting patterns of divergence at the regulatory and sequence level in European Daphnia galeata natural populations
Source: Ecol Evol. 2019 Feb 12;9(5):2487–504. doi: 10.1002/ece3.4894 (PMC6405927; doi:10.1002/ece3.4894)

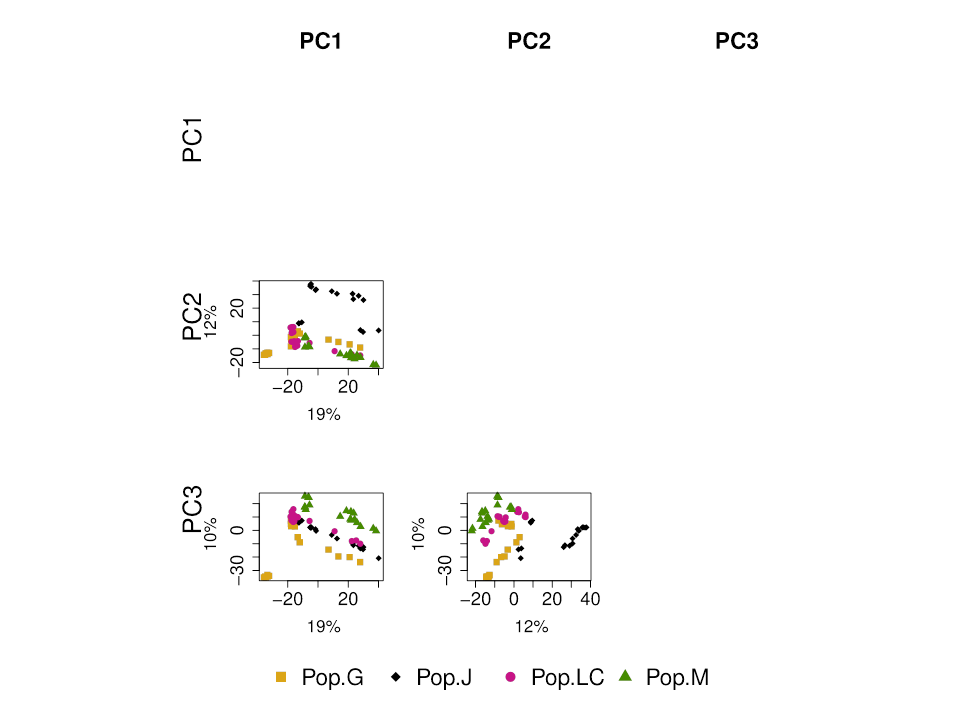

Supplement: Supplementary file 3 [file ECE3-9-2487-s003.tif]

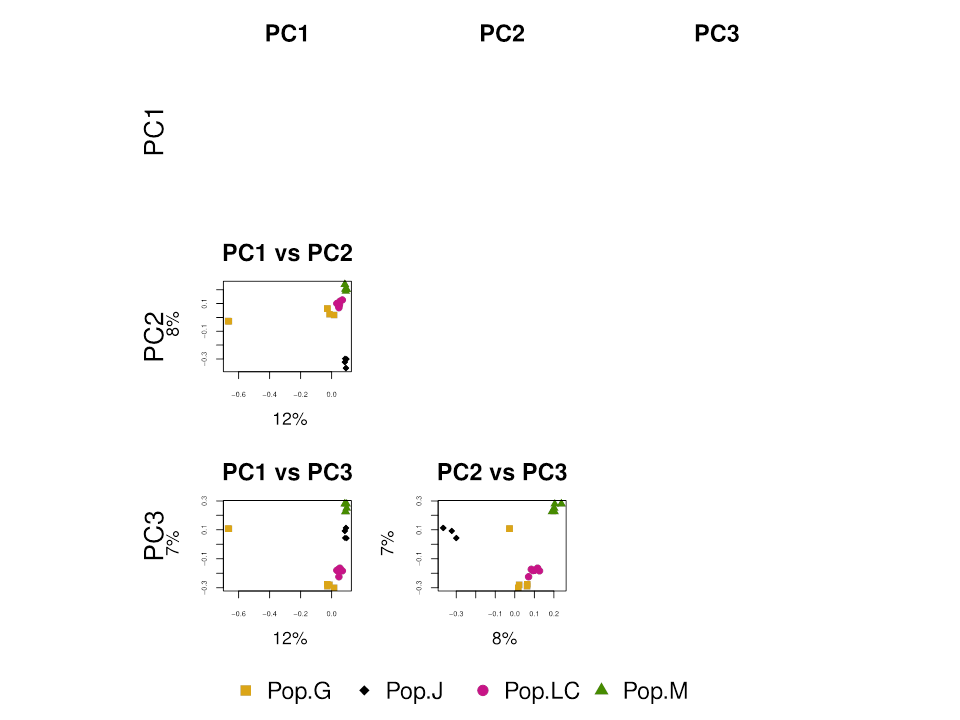

Supplement: Supplementary file 4 [file ECE3-9-2487-s004.tif]

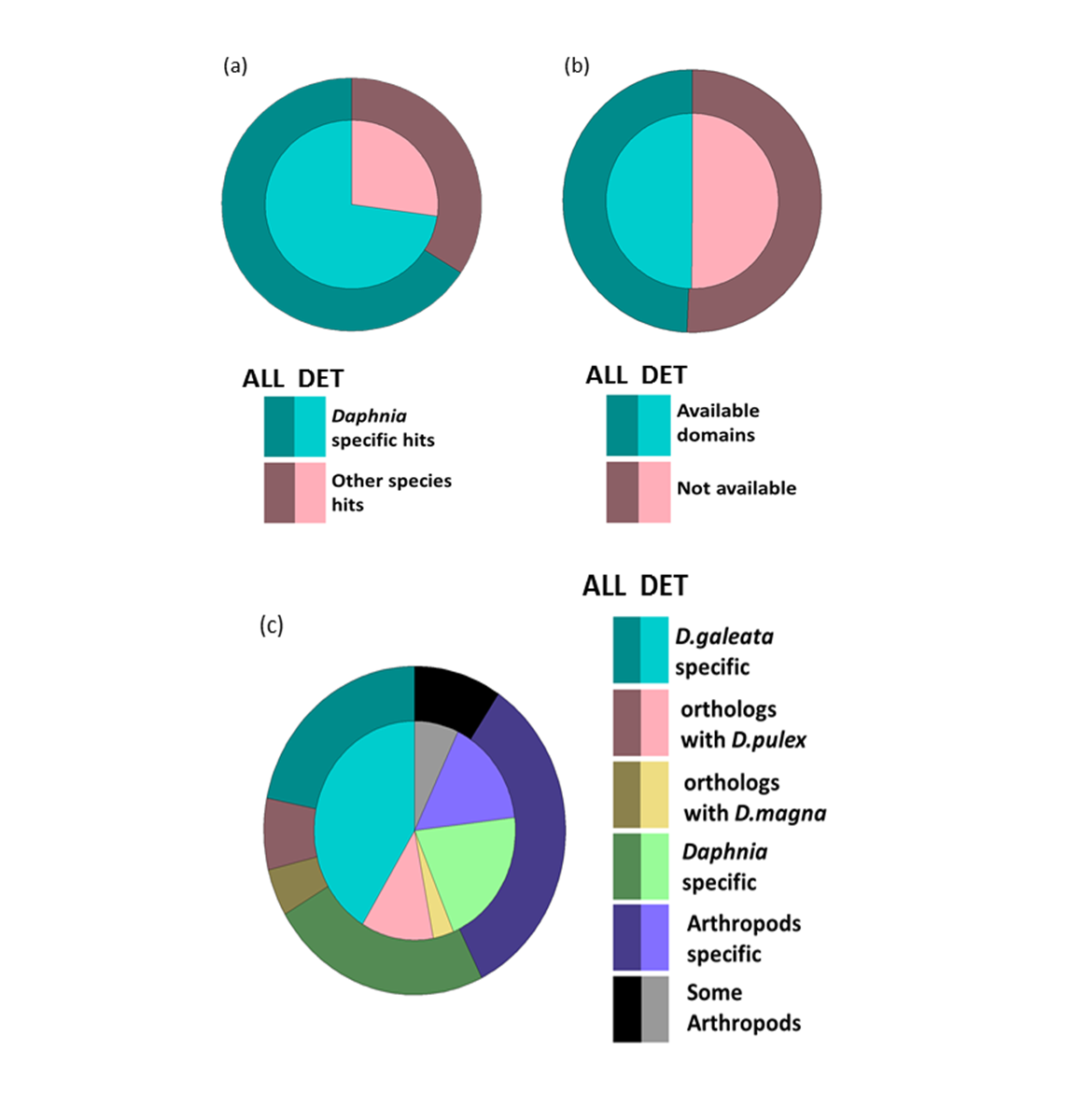

Supplement: Supplementary file 5 [file ECE3-9-2487-s005.tif]
